# Pulmonary Alveolar Microlithiasis

**DOI:** 10.1155/2016/4938632

**Published:** 2016-03-31

**Authors:** Kevan Mehta, Sharon Dell, Catherine Birken, Suhail Al-Saleh

**Affiliations:** Hospital for Sick Children, 555 University Avenue, Toronto, ON, Canada M5G 1X8

## Abstract

Pulmonary alveolar microlithiasis (PAM) is a rare autosomal recessive condition that is often asymptomatic despite significant changes in chest imaging. Diagnosis is often made when patients become symptomatic in adulthood. There are still no proven treatments, but earlier diagnosis may allow for evaluation of preventative strategies that could improve outcome. It is an important diagnosis to consider in children who have marked radiographic findings with no or very mild symptoms or physical findings. Diagnosis can be made with imaging alone but may necessitate lung biopsy for definitive diagnosis.

## 1. Case Report

**Figure 1:**
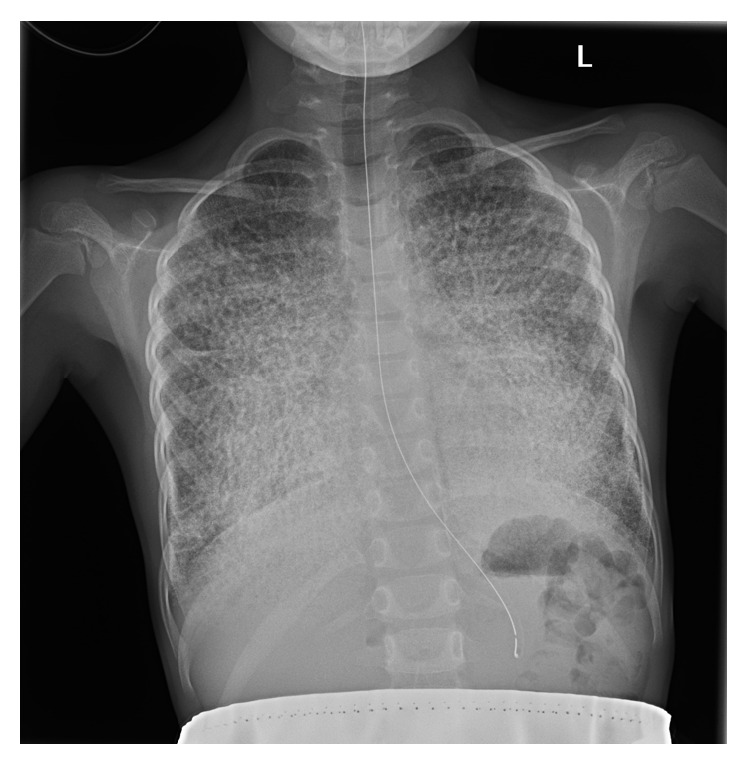
PA chest X-ray.

A 5-year-old girl presented with an acute onset fever, for about 4 days. She was seen initially in her local emergency room and a chest X-ray was done to rule out pneumonia. This showed a marked, diffuse reticulonodular pattern of parenchymal opacity bilaterally (Figure 1), incongruous with her clinical appearance. In reviewing the history, she was well until about 4 days before her presentation with a dry cough, night sweats, and daily fevers. During this time, she had no other significant respiratory symptoms, including no chest pain, no shortness of breath, no tachypnoea, and no haemoptysis.

Her medical history included a possible episode of pneumonia at one year of age, diagnosed and treated as an outpatient. A chest X-ray done at this time showed the beginning of a reticulonodular pattern that was considered to be due to infection and not followed up, as she recovered and remained clinically well thereafter. She had no hospital admissions or surgical procedures done. She received all of her immunizations. She had received salbutamol and fluticasone inhalers for an episode of wheezing at 3 years of age but had not used these thereafter. She had no atopic features. She was born premature, at 32 weeks, and required NICU admission for 2 weeks to establish feeding; there were no reported respiratory issues during the admission. She has a twin sister who has had no medical concerns. The family history was noncontributory but the parents were of Middle-Eastern descent and consanguineous, being first cousins. The patient had not travelled outside of the country and there was no obvious tuberculosis contact.

She had normal vital signs and normal oxygen saturations in room air (heart rate 100/min, respiratory rate 22/min, saturation 97%, temperature 36.1 degrees Celsius, and blood pressure 90/51 mmHg). Her height and weight were on the 3rd percentile. She had mildly increased work of breathing but was otherwise comfortable at rest. Her chest had bibasal inspiratory crepitations, bronchial breath sounds in the midzones bilaterally, and clear upper zones. Her cardiovascular, gastrointestinal, and head/neck examination was normal.

Her nasopharyngeal swab was found to be positive for influenza A. Her complete blood count was unremarkable with a white cell count of 7.2 × 10^9^/L, haemoglobin of 123 g/L, and platelets of 223 × 10^9^/L. Her inflammatory markers were done, with a C-reactive protein of 15.9 mg/L and an erythrocyte sedimentation rate of 41 mm/hr. Immunoglobulins were sent showing a normal IgG of 10.5 g/L, normal IgM of 1.9 g/L, and slightly elevated IgA of 2.4 g/L. She had normal electrolytes, including normal serum calcium and magnesium. Her liver enzymes were also normal.

A wide differential that included rheumatological, infectious, oncological, respiratory, and immunological causes was considered and so multiple investigations were then arranged. She had gastric aspirates and a tuberculin skin test to rule out tuberculosis, all of which were normal. Autoantibodies were sent and she was found to be mildly antinuclear antibody (ANA) positive (titres of 1 : 160) but her other autoantibodies were negative. She had adenine deaminase (ADA) levels and lymphocyte immunophenotyping done to rule out immune deficiency, which were normal. She had a normal sweat chloride. Bronchoscopy with bronchoalveolar lavage (BAL) was done: there was no bacterial or fungal growth on culture and the fluid was normal in appearance; BAL cell count showed WBC 127 × 10^6^/L (3% neutrophils, 2% lymphocytes, and 95% macrophages) and RBC 303 × 10^6^/L.

**Figure 2:**
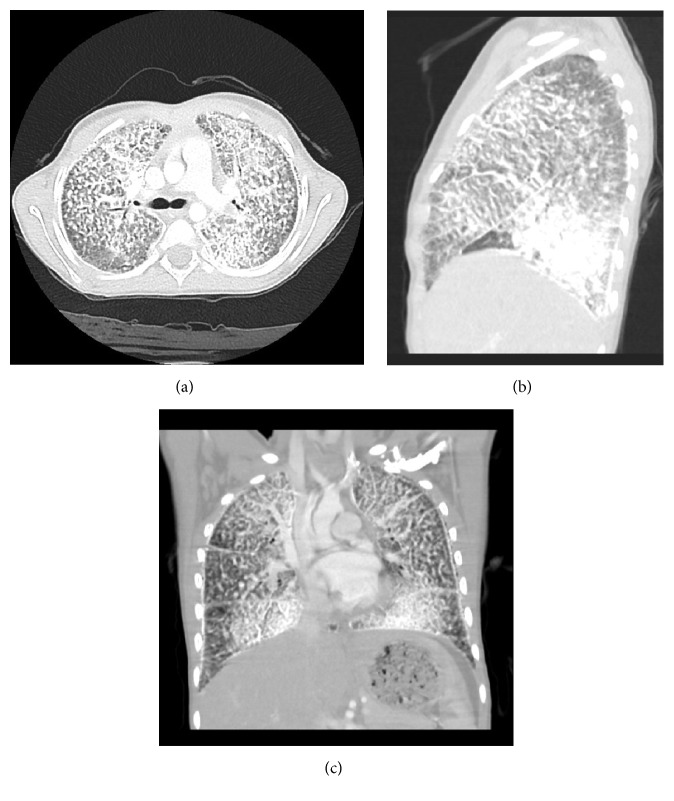
CT. (a) Axial CT image. (b) Sagittal CT image. (c) Coronal CT image.

In regard to other imaging, she had a normal abdominal and thyroid ultrasound. Her chest CT showed diffuse interstitial lung disease, essentially characterized by interstitial lobular septal thickening (Figures 2(a), 2(b), and 2(c)). This raised possible diagnoses of pulmonary alveolar microlithiasis, dendritic pulmonary ossification (DPO), and pulmonary alveolar proteinosis (PAP).

**Figure 3:**
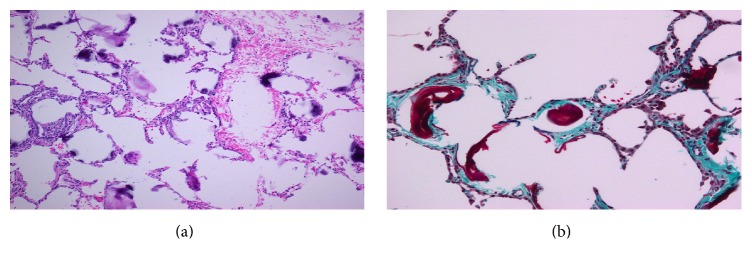
Histopathology. (a) Hematoxylin and eosin stain (×10). (b) Trichrome stain (×20).

As the imaging was not definitive, a lung biopsy was done; tissue was obtained from the lingula, which was sent for microbiology and pathology. The microbiology was negative. Pathology demonstrated presence of calcium concretions within the alveolar spaces, with blue discoloration at the edges and an eosinophilic centre on hematoxylin and eosin (H&E) stain; some structures had a concentric lamellar arrangement on trichrome staining (Figures 3(a) and 3(b)). These findings were consistent with the diagnosis of pulmonary alveolar microlithiasis, with an incidental influenza infection explaining her acute symptoms.

The patient was then discharged home after recovery from the biopsy. Genetic testing showed homozygous pathogenic mutations in the SLC34A2 gene (NM_006424.2:c.226C>T [p.Gln76∗] in exon 03). Her sister was then brought for chest X-ray and CT, which showed similar findings, and was also positive on genetic testing.

## 2. Discussion

Pulmonary alveolar microlithiasis is a rare autosomal recessive disease, where there is formation of intra-alveolar calcium phosphate microliths, building up gradually over time. Its true prevalence is unknown with about 500 case reports in the literature; only about one-third of these are in patients under twenty years of age [1]. There appears to be a higher prevalence in Asia Minor and Europe, with many case reports in Turkish patients [2]. Mutations in the SLC34A2 gene cause loss of function in the sodium-phosphate transporter type IIb. This is thought to result in phosphate accumulation within the alveoli, creating a nidus for microlith formation. Although the true sensitivity of genetic testing is yet to be determined, due to the rare nature of the disease, in the few patients in the literature that have had genetic testing, all have tested positive for mutations in the SLC34A2 gene [3]. Patients are usually remarkably asymptomatic, with the most typical feature being “clinical radiological dissociation,” where an X-ray shows a significant “sandstorm” appearance despite a paucity of symptoms or examination findings. Symptoms typically appear in the third or fourth decade of life; most common is dyspnoea and dry cough but other symptoms include chest pain, haemoptysis, fatigue, and pneumothoraces [4]. Generally microlith formation is localized to the alveoli, but there are case reports of microliths in other structures including gallbladder, urethra, seminal vesicles, kidneys, pleura, aortic valve, and/or arteries [4]. There are associations with pectus excavatum, hypertrophic pulmonary osteoarthropathy, milk-alkali syndrome, diaphyseal aclasis, autosomal recessive Waardenburg anophthalmia syndrome, and lymphocytic interstitial pneumonitis [4]. Diagnosis is often considered in the third or fourth decade, when symptoms begin to appear but can be diagnosed in childhood, with the youngest reported patient being diagnosed at 8 months of age [5]. It is often diagnosed by chest X-ray and a CT scan, the latter of which shows “crazy paving” pattern, with combination of ground glass opacities and thickened interlobular septa; there may also be a “black pleura” sign, with there being subpleural cysts separated from underlying calcified lung parenchyma. Definitive diagnosis is with lung biopsy, typically reserved for cases where there is uncertainty, although if the clinical picture is consistent, one can arguably send for genetic testing first to avoid this invasive procedure. Biopsy shows intra-alveolar concretions, seen best with trichrome staining, which helps to differentiate it from DPO, which typically has metaplastic osteoblast formation and is preferentially in the interstitium as opposed to the alveolar spaces; furthermore, DPO was less likely in this case as it is often associated with chronic pulmonary or systemic disease and has not been described with mutations in the SLC34A2 gene. Pulmonary function is usually normal in the asymptomatic state, eventually progressing to a restrictive picture. BAL can be helpful if microliths are recovered but otherwise is usually normal. The only truly effective treatment to date is lung transplant with several case reports of individuals being asymptomatic 1, 5, 10, and even 15 years from transplant [6]. Repeated therapeutic BAL and steroids have not been proven to be effective [7, 8]. There is ambivalent evidence to support disodium etidronate therapy, with several reports of radiographic improvement but others showing no significant change [9]. These patients must be followed up as they invariably develop respiratory failure, often within 10–15 years of their diagnosis, and are candidates for lung transplantation.

## Additional Points

*Learning Objectives*. Identify clinical and radiological features of pulmonary alveolar microlithiasis in children.

*Royal College of Physicians and Surgeons of Canada CanMEDS Roles.* This article addresses the Medical Expert and Scholar roles.

*Pretest Questions*

- When should you consider the diagnosis of pulmonary alveolar microlithiasis?
- What are the investigations required to diagnose pulmonary alveolar microlithiasis?

*Posttest*

(i) When should you consider the diagnosis of pulmonary alveolar microlithiasis?
 The diagnosis should be considered in any individual, who has significant radiographic changes with no or very few symptoms and physical findings. The X-ray often has a typical “sandstorm” appearance and the CT will usually show a “crazy paving” pattern.
(ii) What are the investigations required to diagnose pulmonary alveolar microlithiasis?
 Usually, a thorough history and physical that is benign, in combination with a chest X-ray and CT scan, will be adequate to diagnose a patient. Bronchoalveolar lavage may be useful if microliths are obtained and pulmonary function may show a restrictive pattern but could be normal. Lung biopsy can be undertaken in cases with atypical features and should definitively establish the diagnosis. Alternatively, genetic testing can be sent prior to biopsy, knowing that its exact sensitivity as a diagnostic test is yet to be established but, if positive, is usually diagnostic.
